# Histone Acetylation Regulator-Mediated Acetylation Patterns Define Tumor Malignant Pathways and Tumor Microenvironment in Hepatocellular Carcinoma

**DOI:** 10.3389/fimmu.2022.761046

**Published:** 2022-01-25

**Authors:** Yuyan Xu, Wei Liao, Qiong Luo, Dinghua Yang, Mingxin Pan

**Affiliations:** ^1^ General Surgery Center, Department of Hepatobiliary Surgery II, Guangdong Provincial Research Center for Artificial Organ and Tissue Engineering, Guangzhou Clinical Research and Transformation Center for Artificial Liver, Institute of Regenerative Medicine, Zhujiang Hospital, Southern Medical University, Guangzhou, China; ^2^ The Unit of Hepatobiliary Surgery, The General Surgery Department, Nanfang Hospital, Southern Medical University, Guangzhou, China; ^3^ Department of General Surgery, Affiliated Hengyang Hospital, Southern Medical University (Hengyang Central Hospital), Hengyang, China

**Keywords:** histone acetylation, tumor microenvironment, hepatocellular carcinoma, drug sensitivity, immunotherapy

## Abstract

**Background:**

Histone acetylation modification is one of the most common epigenetic methods used to regulate chromatin structure, DNA repair, and gene expression. Existing research has focused on the importance of histone acetylation in regulating tumorigenicity, tumor progression, and tumor microenvironment (TME) but has not explored the potential roles and interactions of histone acetylation regulators in TME cell infiltration, drug sensitivity, and immunotherapy.

**Methods:**

The mRNA expression and genetic alterations of 36 histone acetylation regulators were analyzed in 1599 hepatocellular carcinoma (HCC) samples. The unsupervised clustering method was used to identify the histone acetylation patterns. Then, based on their differentially expressed genes (DEGs), an HAscore model was constructed to quantify the histone acetylation patterns and related subtypes of individual samples. Lastly, the relationship between HAscore and transcription background, tumor clinical features, characteristics of TME, drug response, and efficacy of immunotherapy were analyzed.

**Results:**

We identified three histone acetylation patterns characterized by high, medium, and low HAscore. Patients with HCC in the high HAscore group experienced worse overall survival time, and the cancer-related malignant pathways were more active in the high HAscore group, comparing to the low HAscore group. The high HAscore group was characterized by an immunosuppressive subtype because of the high infiltration of immunosuppressive cells, such as regulatory T cells and myeloid-derived suppressor cells. Following validation, the HAscore was highly correlated with the sensitivity of anti-tumor drugs; 116 therapeutic agents were found to be associated with it. The HAscore was also correlated with the therapeutic efficacy of the PD-L1 and PD-1 blockade, and the response ratio was significantly higher in the low HAscore group.

**Conclusion:**

To the best of our knowledge, our study is the first to provide a comprehensive analysis of 36 histone acetylation regulators in HCC. We found close correlations between histone acetylation patterns and tumor malignant pathways and TME. We also analyzed the therapeutic value of the HAscore in targeted therapy and immunotherapy. This work highlights the interactions and potential clinical utility of histone acetylation regulators in treatment of HCC and improving patient outcomes.

## Introduction

Hepatocellular carcinoma (HCC) is the most common primary liver cancer and ranks as the fifth leading malignancy worldwide (1). Most patients with HCC have poor outcomes because of limited early diagnosis and few available treatment options for advanced-stage HCC (2). Even with active treatment, such as liver transplantation, resection, percutaneous ablation, transarterial chemoembolization, HCC is likely to recur and metastasize, with a 5-year survival rate of less than 20% (3, 4). In addition, both traditional chemotherapy and molecular-targeted agents are impeded by tumor heterogeneity, as well as the intrinsic and acquired drug resistance that can develop in tumors. These characteristics limit the efficacy of systemic therapy in HCC patients (5). Therefore, there is an urgent need to investigate new strategies to improve the clinical outcomes of patients with HCC. Recently, with deeper exploration of the relationship between the immune system and cancer, new therapeutic strategies aimed at mobilizing the host immune system to eradicate tumor cells would advance the cancer therapy field and introduce greater efficacy in curing cancer.

Numerous cancer immunotherapy strategies have rapidly emerged in recent years. The most notable immune-checkpoint inhibition (ICI) treatments consist of agents targeting the inhibitory immune receptors, cytotoxic T-lymphocyte (CTL)-associated protein 4 (CTLA-4/CD152), programmed death protein 1 (PD-1/CD279), and programmed death ligand 1 (PD-L1/B7H1/CD274). These agents have become effective standard therapies in several advanced malignancies, including melanoma (6–8), Merkel cell carcinoma (9), urological cancers (10), non-small cell lung cancer (11), mis-match repair-deficient tumors (12), and Hodgkin’s lymphoma. Their response rates range from 25 to 60% in first- and second-line settings (13). Recently, ICI treatment has also been approved for HCC, gastric cancer, triple negative breast cancer, cervical cancer, and head and neck cancer, with response rates closer to 15% (14).

Nonetheless, the efficacy of ICI treatment is still limited because of the ability of cancer tumors to develop primary, adaptive, or acquired resistance to immunotherapy. The resistance of cancer to immunotherapy depends on various factors including the tumor microenvironment (TME), the patient’s genetic background, epigenetics, metabolism, and cell stemness (15). At the same time, the multiple factors involved in immunotherapy resistance also provide many more targets that can be attacked by therapeutic agents. To improve the efficacy of immunotherapy, ICI can be combined with other treatments to overcome the immunotherapy resistance.

One such treatment involves histone acetylation. This is one of the most common epigenetic methods used to regulate chromatin structure, DNA repair, and gene expression (16). Histone acetylation is a type of posttranslational modification in which multiple lysine residues at the N-terminus of histones are catalyzed by histone acetyltransferases (HATs). This process is highly dynamic, reversible, and regulated by proteins that can be divided into three categories: “writer”, “reader”, and “eraser”. The “writers” refer to enzymes that transfer acetyl groups to histones, and the “erasers” refer to enzymes that remove acetyl groups from histones. The “readers” are effector proteins that can recognize the modified histones (17). Acetylation neutralizes the positive charge on lysine, weakening the electrostatic association between the histones and the DNA; this makes the DNA becomes more accessible to transcription factors (18).

In general, histone acetylation is associated with elevated transcription whereas histone deacetylation is often associated with gene repression. Previous reports have demonstrated that histone acetylation is closely related to tumorigenesis and can impact certain biological processes of tumor cells, including proliferation (19), apoptosis (20), metastasis (21), and stemness (22). Histone deacetylases (HDACs) are critical regulators of gene expression that enzymatically remove acetyl groups from histones. As such, they are an example of “erasers.” Numerous correlative studies have demonstrated aberrant expression of HDACs (HDAC1, HDAC5, and HDAC7) in human tumors, which can serve as molecular biomarkers to distinguish between tumorous and normal tissue (23). HDAC inhibitors (HDACi) can induce acute hyperacetylation of histones and generate the re-expression of tumor-suppressor genes to inhibit tumor growth. Many HDACi have been proven to have potent anti-tumor effects in several hematological and solid malignancies (24, 25). Recently, researchers have found that histone acetylation is closely related to the TME. Furthermore, numerous studies have demonstrated that HDACi can reshape the TME *via* various mechanisms, enhancing the ability of the immune system to kill tumor cells. Specifically, these mechanisms include upregulating the expression of tumor antigens, enhancing antigen-processing ability, improving the cytolytic activity of CD8+ T cells, and disrupting the immunosuppressive function of IL-10 producing regulatory T cells (26–29). For instance, in preclinical cancer models, HDACi were shown to enhance the efficacy of immune checkpoint blockade using anti-PD1/PDL1 or anti-CTLA4, immunostimulant therapies such as anti-CD40 and anti-CD137, and adoptive T cell immunotherapy (30–34).

Collectively, the above findings indicate that histone acetylation plays an important role in the regulation of the TME, and the molecular agents that target histone acetylation regulators have the potential to disrupt cancer immunotherapy resistance. As a result, combining molecular agents that target histones with immunotherapy could produce additional clinical benefit to patients. However, due to limitations in technical methodology, previous analysis has been confined to a small number of histone acetylation regulators, whereas the antitumor effect of histone acetylation modification is characterized by highly integrated interactions of numerous regulators. Therefore, a comprehensive understanding of how the regulatory network of multiple histone acetylation regulators affects the biological behavior of tumor cells and TMEs would contribute to the development of immunotherapeutic strategies.

In this study, we retrospectively investigated genomic alterations in 1599 HCC samples from the Cancer Genome Atlas (TCGA), International Cancer Genome Consortium (ICGC), and Gene Expression Omnibus (GEO) cohorts. Our objective was to comprehensively evaluate the patterns of histone acetylation modification based on 36 histone acetylation regulators. We found that histone acetylation patterns are distinct in their activation of malignant cancer-related pathways and infiltration of multiple immune cells. We also constructed an HAscore model to quantify the histone acetylation patterns in individual patients based on the differentially expressed genes (DEGs) among them. Finally, we assessed the therapeutic value of the HAscore in targeted HCC therapy and immunotherapy.

## Materials And Methods

### Collection of HCC Datasets and Preprocessing

The workflow of the study is shown in **Figure S1A**. Gene expression data and clinical features of liver cancer samples were retrospectively retrieved from publicly available datasets of the NCBI GEO database (https://www.ncbi.nlm.nih.gov/geo/), TCGA (https://portal.gdc.cancer.gov/), and ICGC (https://dcc.icgc.org/). Specifically, the clinical data we used from the TCGA database included tumor stage, histological grade, vascular tumor cell type, viral hepatitis serologies, Child–Pugh scores, alpha-fetoprotein (AFP), gender, and overall survival (OS) times. In addition, we obtained genomic mutation data (including somatic mutation and copy number variation) of TCGA-LIHC from the UCSC Xena database. In general, nine hepatocellular carcinoma cohorts—TCGA-LIHC, ICGC-LIRI (Japan), ICGC-LICA (France), GSE14520, GSE76427, GSE116174, GSE104580, GSE112790, and GSE121248—for 1599 patients were included for further analysis.

RNA sequencing data, including fragments per kilobase million (FPKM) values and count values, were consistently transformed into transcripts per kilobase million (TPM) values (35). For microarray data from GEO, the normalized matrix files were directly downloaded and normalized by the “normalizeBetweenArrays” method of the R package limma after gene symbol transformation, so that the intensities or log-ratios would have similar distributions across a set of arrays (36). Finally, we used the “ComBat” method of the sva Package (37) to adjust the batch effect caused by non-biotechnological bias.

Two immune checkpoint blockade treatment cohorts with available expression and clinical information were used in our study. First, we obtained the IMvigor210 cohort (http://research-pub.gene.com/IMvigor210CoreBiologies), which consists of advanced urinary tract transitional cell carcinoma treated with atezolizumab, an anti-PD-L1 antibody (38). Second, we obtained the *David Liu* cohort (https://www.nature.com/articles/s41591-019-0654-5), which consists of metastatic melanoma treated with nivolumab or pembrolizumab (39). The gene expression profiles of the pre-therapy biopsy samples were curated and transformed into the TPM format for further analysis.

We searched and collected the following datasets with targeted therapy and chemotherapy from the GEO database: the GSE5851 dataset (advanced metastatic colorectal cancer treated with cetuximab monotherapy); GSE148623 dataset (ductal breast cancer treated with ricolinostat, an HDAC6 inhibitor); and GSE22219 dataset (early primary breast cancer treated with adjuvant cyclophosphamide, methotrexate, and 5-fluorouracil).

Corresponding clinical data were collected from the appropriate GEO dataset metadata and the supplemental files of relevant articles. All baseline information on the available data is summarized in **Table S1**.

### Consensus Clustering Expression Pattern of 36 Histone Acetylation Regulators

The literature related to histone acetylation modification was retrieved, and 36 acknowledged histone acetylation genes were curated and analyzed to identify distinct histone acetylation modification patterns (**Table S2**). An unsupervised consensus clustering algorithm was applied to determine robust clustering of liver cancer. We used the R package ConsensusClusterplus to perform the above steps and conducted 1000 repetitions to ensure the stability of the classification (40).

### Gene Set Variation Analysis (GSVA) and Functional Annotation

To explain the differences in biological processes between histone acetylation modification patterns, we realized GSVA enrichment analysis by using “GSVA” R packages. This method is commonly used to estimate the variation in pathways and biological process activity in samples of an expression dataset (41). The gene sets of “h.all.v7.4.symbols” were downloaded from the MSigDB database for further GSVA analysis. The 13 most common oncogenic hallmarks, epithelial-to-mesenchymal transition (EMT), and cancer stem cell (CSC) signatures were obtained from the supplementary table prepared by Sanchez-Vega et al. (**Table S3**) (38, 42, 43). Differences were considered statistically significant at *P* values < 0.05. We used the clusterProfiler R package to perform functional annotation for histone acetylation modification-related genes, with a cutoff value of FDR < 0.05 (44).

### Estimation of TME Cell Infiltration

We used the single-sample gene-set enrichment analysis (ssGSEA) algorithm to quantify the relative abundance of each cell infiltration in the HCC TME. The gene sets defining each immune cell type were obtained from the study by Charoentong (**Table S4**) (45). The enrichment scores calculated by ssGSEA analysis were used to represent the relative abundance of the TME infiltrating cells in each sample. The immune-related features were collected from previously published studies (**Table S3**) (46, 47).

### Differentially Expressed Genes (DEGs) Among Histone Acetylation Modification Phenotypes

To identify histone acetylation modification-related genes, we classified patients into three distinct histone acetylation modification patterns based on the expression of the 36 histone acetylation modification regulators. DEGs among different modified histone acetylation patterns were determined using limma (36). The significance criteria for determining DEGs were set as adjusted *P* values < 0.001 and |FC| > 1.5. The adjusted *P* value for multiple testing was calculated using the Benjamini–Hochberg correction.

### Construction of Histone Acetylation Gene Signatures

To quantify the modified histone acetylation patterns of individual tumors, we developed a scoring scheme to quantify the histone acetylation modification level of individual patients and described it as the HAscore. Specifically, 965 DEGs were first identified from different HAclusters, and prognostic analysis was performed for the DEGs using univariate Cox regression model analysis. Subsequently, 591 genes with significant prognoses were selected for further analysis. Next, the patients were classified into several groups for further analysis by adopting an unsupervised clustering method for analyzing prognosis-related DEGs. The consensus clustering algorithm was used to define the number of gene clusters and their stability. We then transformed the expression of these genes into a Z score and conducted principal component analysis (PCA) to construct modified acetylation-relevant gene signatures. Both principal components 1 and 2 (PC1 and PC2, respectively) were selected to act as signature scores. This method focused on the score of the set with the largest block of well-correlated (or anti-correlated) genes, while down-weighting contributions from genes that did not track with other set members. We then adopted a formula like that of previous studies to define the HAscore (48, 49):


HAscore=Σ(PC1i+PC2i)

where i is the expression of histone acetylation modification phenotype-related genes

### Calculation of the EMT Score

EMT gene signatures were collected from Mak et al. (50), including 25 epithelial and 52 mesenchymal marker genes. Similar to this previous study (50, 51), the EMT score for each sample was evaluated as $\Sigma_{i}^{N}\frac{M^{i}}{N}-\Sigma_{j}^{n}\frac{E^{j}}{n}$ , where M and E represent the expression of the mesenchymal and epithelial genes, respectively. Likewise, N and n represent the number of mesenchymal and epithelial genes, respectively.

### Correlation Analysis of HAscore and Drug Sensitivity

The Genomics of Drug Sensitivity in Cancer (GDSC) database is the largest public resource for information on drug sensitivity in cancer cells and molecular markers of drug response (52). From here, we collected the transcription profiles of approximately 1000 cancer cell lines, drug response measurements (as AUC of the drug-sensitive curve) in cancer cell lines, as well as targets and pathways of drugs. We performed Spearman correlation analysis to calculate the correlation between drug sensitivity and HAscore and considered |Rs| > 0.3 and FDR < 0.05, estimated by Benjamini and Hochberg adjustment, as significant correlation.

### Quantification of the Immune Response Predictor: TIDE

The tumor immune dysfunction and exclusion (TIDE) algorithm proposed by Jiang et al. was used to predict immune checkpoint blockade response by modeling distinct tumor immune evasion mechanisms, including the induction of T cell dysfunction in tumors with high infiltration of CTL and the prevention of T cell infiltration in tumors with low CTL levels by immunosuppressive cells (53). A higher TIDE score indicates that tumor cells are more likely to induce immune escape, thus indicating a lower response rate to ICI treatment. In our study, we used the all-sample average in each study as the normalization control and calculated the TIDE score of each sample using the TIDE tool on the TIDE web application (http://tide.dfci.harvard.edu/), following the developer’s instructions.

### Statistical Analysis

The data were analyzed using R (version 4.0.0) and R Bioconductor packages. The normality and homogeneity test of variance were tested using the Shapiro–Wilk normality test and Bartlett homogeneity test, respectively. The Wilcoxon test, Kruskal–Wallis test, and t-test or one-way ANOVA were used to compare the differences as nonparametric or parametric methods. Correlation coefficients were computed using Spearman’s and distance correlation analyses. A receiver operating characteristic (ROC) curve was used to verify the validity of the model. Based on the correlation between HAscore and patient survival, the Survminer package was used to determine the best cutoff point of survival information for each cohort. The surv-cutpoint function was used to dichotomize the HAscore, and all potential cutting points were repeatedly tested to find the maximum rank statistic. Then, the patients were divided into high and low HAscore groups according to the maximum selected log-rank statistics to lessen the calculated batch effect. Survival curves for the prognostic analysis were conducted using the Kaplan–Meier method, and log-rank tests were used to assess differences between groups. The chi-squared test or Fisher test was used to analyze the differences in clinical features between the HAscore groups. A univariate Cox regression model was used to generate the hazard ratio (HR) for histone acetylation regulators and histone acetylation-related genes. To verify whether the HAscore was an independent prognostic predictor, we incorporated the HAscore and related clinical parameters into a multivariate Cox regression model analysis. All statistical analyses were two-sided, and statistical significance was set at *P* < 0.05.

## Results

### Genetic and Transcriptional Alterations of the 36 Histone Acetylation Regulators in HCC

**Figure 1:**
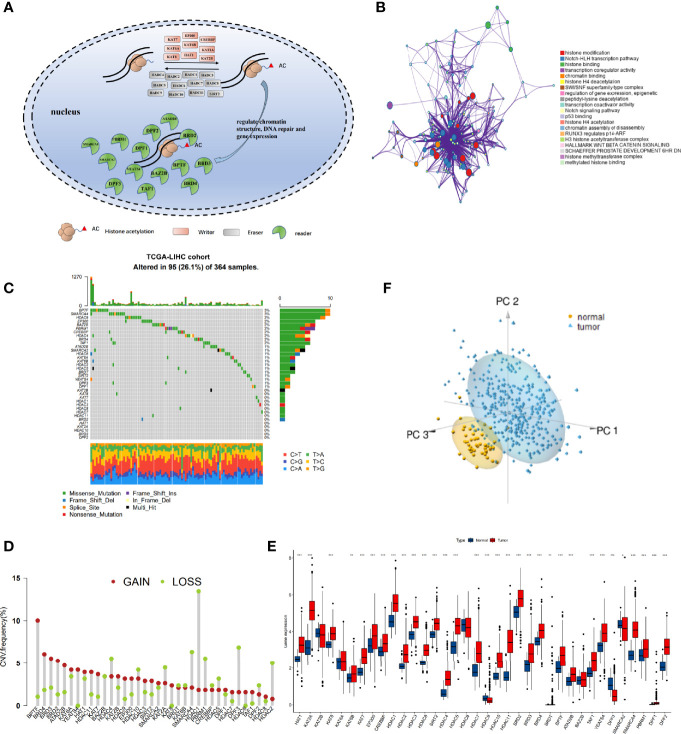
The landscape of genetic alterations of histone acetylation regulators in hepatocellular carcinoma (HCC). **(A)** Summary of the dynamic reversible process of histone acetylation modification mediated by regulators (“writers,” “erasers,” and “readers”) and their biological functions. **(B)** Functional annotations of 36 regulators analyzed by the Metascape enrichment tool. Cluster annotations are shown in the color code. **(C)** The mutation frequency of 36 histone acetylation regulators in TCGA-LIHC cohort. Each column represents individual patients. The barplot on top shows TMB, and the numbers on the right display the mutation frequency of each regulator. The barplot on the right shows the proportion of each variation type. The stacked barplot on the bottom displays the fraction of conversions in each sample. **(D)** The copy number variation (CNV) frequency of histone acetylation regulators in TCGA-LIHC was prevalent. The column represents the alteration frequency. The deletion frequency is a light-green dot; the amplification frequency is a crimson dot. **(E)** Boxplot shows the expression of the 36 histone acetylation regulators between tumor and normal tissues in the TCGA-LIHC cohort. Tumor: red; Normal: blue. (*P < 0.05, **P < 0.01, ***P < 0.001). **(F)** Principal component analysis of the 36 histone acetylation regulators to distinguish tumors from normal samples in TCGA-LIHC. Tumor: pale blue; normal: yellow.

After a systematic review of published articles about histone acetylation, 36 histone acetylation regulatory genes in HCC were identified and incorporated into our analysis, including 9 “writers”, 12 “erasers”, and 15 “readers”, as shown in **Figure 1A** (**Table S2**). Metascape analyses and KEGG enrichment of the 36 histone acetylation regulators were conducted. Significantly enriched biological processes were mainly related to histone modification and cancer-related pathways, as summarized in **Figures 1B** and **S1B**. To determine the genetic alterations of histone acetylation regulators in cancer, we assessed the prevalence of non-silent somatic mutations in the 36 histone acetylation regulators. In the HCC cohort of TCGA, 95 of the 364 (26.1%) samples experienced genetic alterations in histone acetylation regulators, primarily involving missense mutations and splice-site mutations (**Figure 1C**). Among them, the mutation frequencies of BPTF and SMARCA4 were the highest (3%), followed by HDAC9, EP300, BAZ2B, PBRM1, CREBBP, HDAC4, BRD4, and TAF1. In addition, the mutation co-occurrence across histone acetylation regulators was examined, and we found that there was a significant mutation co-occurrence relationship between TAF1 and SMARCA4 (**Figure S1C**). Furthermore, we examined somatic copy number variations (CNVs) of the 36 regulators and found that CNV was widespread among them, and CNV gain was the major alteration (**Figure 1D**). The location of CNV alteration of m6 A regulators on chromosomes is shown in **Figure S1D**. To ascertain whether these genetic variations influenced the expression of histone acetylation regulators in HCC patients, we compared the mRNA expression of these regulators between normal and HCC samples (**Figure 1E**). The results revealed that most genes were upregulated in the HCC samples than in the normal samples, excluding HDAC9, DPF3, and SMARCA2. The genes with higher frequency of CNV gain than of CNV loss were more likely to be upregulated in tumors (such as BPTF, BRD4, and YEATS4). However, the gene expression patterns of some regulators in tumor and non-tumor samples were not consistent with CNV alteration. For example, HDAC1 had a higher frequency of CNV loss than of CNV gain, but the mRNA expression of HDAC1 was upregulated in HCC samples. To investigate the discrepancy between CNV values and mRNA expression, we divided the HCC cohort into four groups based on CNV value (HCC samples with CNV gain, CNV loss, non-significant alteration of CNV, and normal samples). We analyzed the mRNA alterations in different groups of 10 regulators whose mRNA expression was not significantly consistent with CNV pattern (**Figure S1E**). The results showed that mRNA expression was higher in the CNV gain group than in the other three groups, and mRNA expression was lower in the CNV loss group than in the CNV gain and non-significant CNV groups. The above analyses indicate that CNV changes play an important role in regulating the expression of histone acetylation regulators. Furthermore, based on the expression of these 36 regulators, we were able to distinguish HCC samples from normal samples (**Figure 1F**).

This analysis demonstrated that the genetic landscape and expression pattern of histone acetylation regulators between HCC and normal samples are highly heterogeneous, indicating that the imbalanced expression of histone acetylation regulators may play a crucial role in the onset and development of HCC.

### Identification of Three Clinical Feature-Related Histone Acetylation Patterns Based on the 36 Regulators

**Figure 2:**
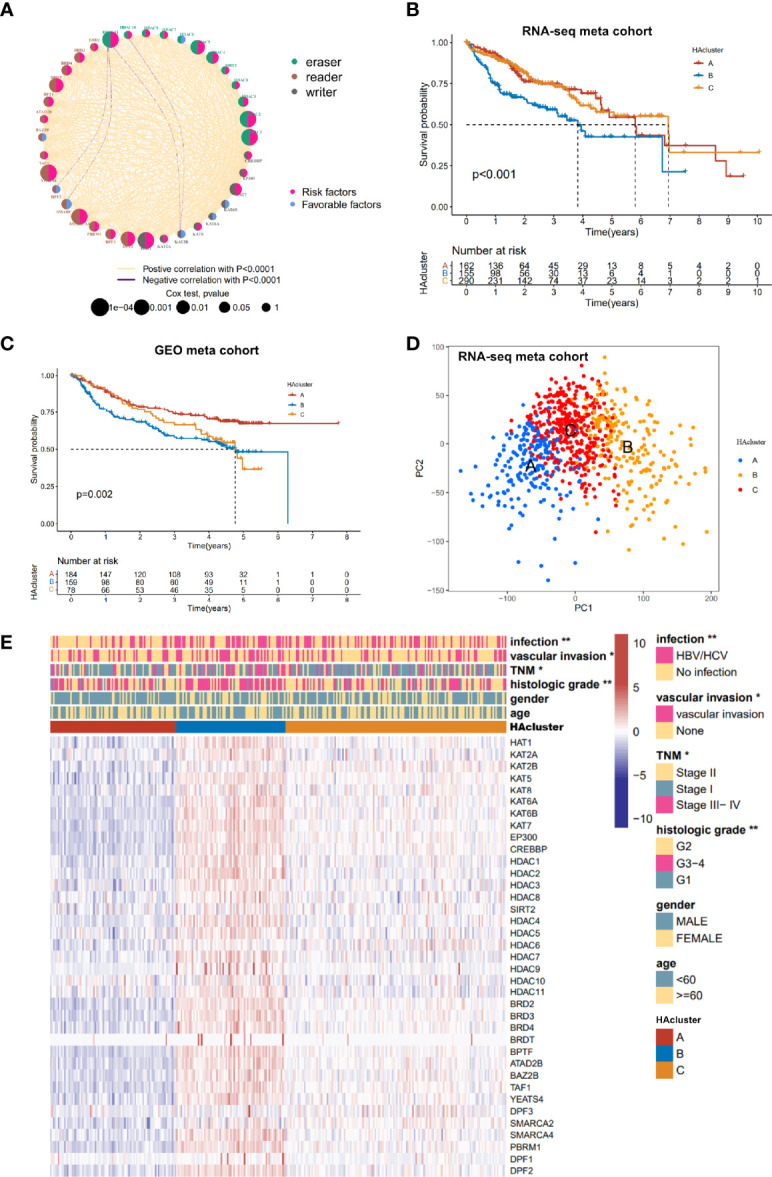
Histone acetylation modification pattern and clinical characteristics of each pattern. **(A)** The interaction among histone acetylation regulators in liver cancer. The circle size describes the effect of each regulator on the prognosis and scale by *P* value. Favorable factors are shown with a pink semicircle on the right. Risk factors are shown with a blue semicircleon the right. Three histone modification types of the 36 histone acetylation regulators are depicted by different colored semicircle on the left. Readers: Indigo; writers: brown; erasers: gray. The red and blue lines represent positive and negative correlations, respectively (*P* < 0.0001). **(B)** Survival analyses of three histone acetylation modification patterns based on 607 patients from the RNA-seq meta cohort (TCGA-LIHC, ICGC-LIRI). **(C)** Survival analyses of three histone acetylation modification patterns based on 421 patients from the GEO meta cohort (GSE14520, GSE76427, GSE116174). **(D)** Principal component analysis of the transcriptome profiles between three histone acetylation modification patterns, indicating a prominent difference on the transcriptome between different HAclusters (based on RNA-seq meta cohort). **(E)** Unsupervised clustering of the 36 histone acetylation modification regulators in the TCGA-LIHC cohort. The HAcluster, viral infection, vascular invasion, TNM stage, histology grade, age, and gender were used as sample annotations. Red represents high expression, and blue represents low expression. Comparison of clinical characteristics proportion analysis between three HAclusters was evaluated by Chi-square test (*P < 0.05, **P < 0.01).

We obtained clinical data and mRNA expression matrices of 1599 HCC samples from nine datasets—TCGA-LIHC, ICGC-LIRI (Japan), ICGC-LICA (France), GSE14520, GSE76427, GSE116174, GSE104580, GSE112790, GSE121248—for further analysis of the expression patterns among the 36 histone acetylation regulators. To explore the prognostic value and expression relationship of histone acetylation regulators, the mRNA sequencing data from the TCGA-LIHC and ICGC-LIRI cohorts with prognostic information were integrated into one meta cohort for univariate Cox regression and Spearman correlation analyses. The results demonstrated that multiple regulators (HDAC2, HDAC1, HAT1, HDAC11, YEATS4, SMARCA4, HDAC5, BRDT, DPF2, HDAC4, KAT7, SMARCA2, BPTF, BRD4, PBRM1, HDAC3, BRD3, DPF1) were risk factors for HCC, and only SMARCA2 was a protective factor against HCC (**Figure S2A** and **Table S5**). Correlation analysis revealed a significant relationship among the expression of the 36 regulators. Most of them were positively correlated with each other, even though they belonged to different biological groups (“writer”, “eraser”, or “reader”) and had different or opposed bio-functions (**Figure S2B**). The expressions of HDAC10 and HDCA11 (“erasers”) were negatively correlated with that of KAT2B (“writer”), and the expression of HDAC11 was negatively correlated with that of DPF3 and SMARC2 (“readers”). These were the only negative correlations between the expressions of the regulators. The comprehensive landscape in the expression network of histone acetylation regulators and their prognostic significance in HCC patients is depicted in **Figure 2A** (**Table S6**). These results indicate that there is a tight cross-talk among the histone acetylation regulators. The writers, erasers, and readers construct a complex network and integrally regulate the histone acetylation modifications, impacting the development of HCC.

To identify the expression pattern of the 36 regulators, the mRNA expression data of 774 HCC samples from the combined datasets (TCGA-LIHC, ICGC-LIRI, and ICGC-LICA cohorts) were classified using ConsensusClusterPlus. Three qualitatively different histone acetylation patterns were identified using unsupervised clustering, including 198 cases in pattern A, 204 cases in pattern B, and 372 cases in pattern C. We termed these patterns HAcluster_A–C (**Figure S2C** and **Table S7**). Clustering of histone acetylation was repeated in the GEO meta cohort (GSE14520, GSE76427, GSE116174, GSE104580, GSE112790, and GSE121248), and a similar result was obtained (**Figure S2D**). Notably, the PCA analysis shows that there was a significant difference in the transcriptional profile among the three different histone acetylation patterns, indicating that unsupervised clustering was successful (**Figure 2D**). The prognostic analysis revealed that the survival probability of patients in HAcluster_B was worse than in HAcluster_A and HAcluster_C based on the combined datasets of TCGA-LIHC and ICGC-LIRI cohorts that have prognostic information (**Figure 2B**). The prognosis predictive ability of the HAcluster was re-examined using the combined data from the GEO database and we obtained similar results (**Figure 2C**). Most histone regulators, including writers, erasers and readers were highly expressed in HAcluster_B, followed by HAcluster_C and HAcluster_A (**Figures 2E** and **S2E**). This indicated that the patients in HAcluster_B have the most active histone acetylation modification and the modification turnover is fast. This may be a risk factor for the prognosis of HCC patients. In addition, the HAcluster was closely correlated with the clinical features of HCC. The viral infection events, vascular invasion, high TNM grade, and high histologic grade were significantly enriched in HAcluster_B, as examined in the TCGA HCC cohort (**Figure 2E**).

### Three Histone Acetylation Patterns Associated With Distinct Tumor Molecular Backgrounds and Immune Infiltration

**Figure 3:**
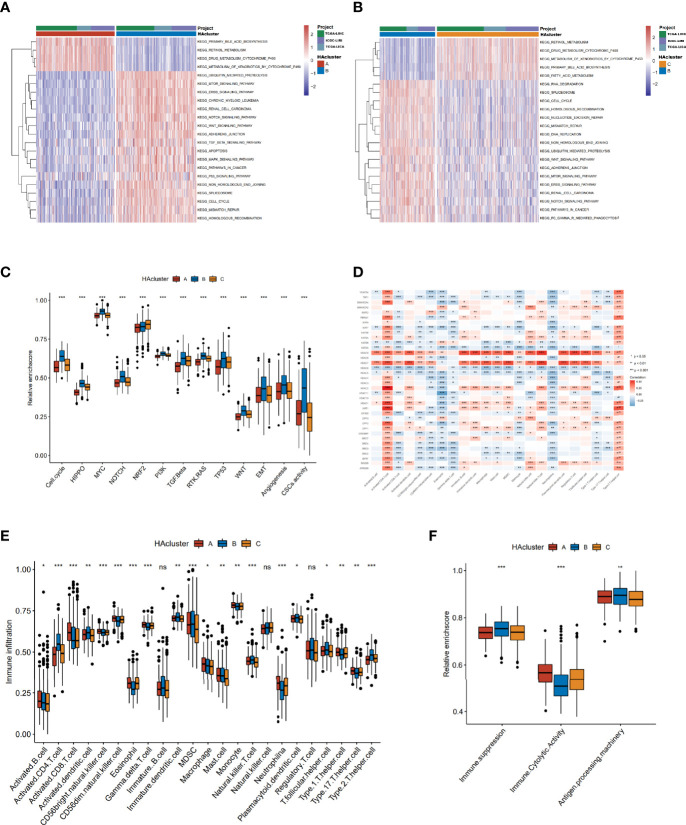
Biological characteristics of histone acetylation patterns. **(A, B)** GSVA enrichment analysis demonstrates the activation states of KEGG biological pathways between distinct HAclusters in RNA-seq meta cohort and the activated group visualized by heatmap. Yellow and blue represent activated and inhibited pathways, respectively. The HAcluster and project of database were used as sample annotations. **(A)** HAcluster A vs HAcluster B; **(B)** HAcluster B vs HAcluster **(C)** Differences in oncogenic pathways among the three distinct HAclusters. **(D)** The correlation between the 36 histone acetylation regulators and TME infiltration cells in RNA-seq meta cohort. Positive and negative correlations are marked in red and blue, respectively. **(E)** Boxplot of abundance of TME-infiltrating cells in three HAclusters, based on the RNA-seq meta cohort. **(F)** Differences in immune-related functional pathways among the three distinct HAclusters. The statistical differences among the three HAclusters were tested by the Kruskal–Wallis test. (*P < 0.05; **P < 0.01; ***P < 0.001; ns, non-significant).

To identify the differences in biological behavior among the three histone acetylation modification patterns, GSVA enrichment analysis based on KEGG gene sets was performed (**Table S8**). Compared to HAcluster_A and HAcluster_C, HAcluster_B was enriched in carcinogenetic activation and stromal pathways, cancer pathways, p53/MAPK/MTOR/NOTCH/WNT/ERBB/TGF_BETA signaling pathways, cell cycle, and apoptosis. On the other hand, HAcluster_A and HAcluster_C were enriched in several biometabolism-related pathways (**Figures 3A, B** and **Table S9**). We confirmed this result by conducting GSVA enrichment analysis based on oncogenic hallmark data obtained by Sanchez-Vega et al. and Mariathasan et al. (**Table S3**) (38, 42); the results showed that HAcluster_B was enriched in most of the malignant pathways, similar to the above analysis (**Figure 3C**). Notably, the activity of angiogenesis, EMT, and cancer stemness was also high in HAcluster_B (**Figure 3C**). As shown in **Figure S3A**, **B**, mRNA expression of stem cell biomarkers in HCC and the EMT score were the highest in HAcluster_B. These analyses indicate that the histone acetylation pattern was closely related to cancer’s bio-behavior in HCC, and the high activity of histone acetylation relators could be a crucial factor in improving the degree of malignancy.

Previous studies have reported a significant correlation between TME infiltration of immune cells and modified histone acetylation (54, 55). Therefore, we comprehensively investigated the functional role of the regulatory network composed of histone acetylation regulators in the TME. The ssGSEA algorithm was used to quantify the relative abundance of immune cells infiltrating the TME (**Table S10**). The Spearman correlation analysis showed a strong correlation between regulators and TME-infiltrating immune cells (**Figure 3D**). For example, the expression of “erasers” HDAC7 and HDAC9 were positively correlated with most of the TME-infiltrating immune cells, and there was a positive correlation between activated CD4 T cells and most of the regulators. Additionally, the differences in TME cell infiltration among thethree histone acetylation patterns were analyzed (**Figure 3E**). HAcluster_B was remarkably differences from HAcluster_A and HAcluster_C. The activated dendritic cells and plasmacytoid dendritic cells were higher in HAcluster_B than in HAcluster_A and HAcluster_C, indicating a highly active antigen-presenting function in this group. The natural killer cells were also high in HAcluster_B. However, activated CD8 T cells, the most powerful effectors in the anticancer immune system (56), along with other important tumor killer cells and gamma delta T cells (57) were both lower in HAcluster B than that in HAcluster_A and HAcluster_C. It is known that myeloid-derived suppressor cells (MDSC) (58) and regulatory T cells are immune suppressive cells (59), while type 2 T helper cells are pro-tumorigenic (60). Both MDSC and type 2 T helper cells were significantly higher in HAcluster_B, and regulatory T cells were higher in HAcluster_B; however, this was not statistically significant. These results indicated that HAcluster_B is an immunosuppressive subtype, and its high levels of immunosuppressive cells offset the positive influence of highly-activated antigen pressing cells, which led to a poor prognosis for patients in HAcluster_B. To confirm this hypothesis, we analyzed the activity of immune suppression, immune cytolytic effect, and antigen processing in the three histone acetylation patterns based on the related gene signature data from Bindea et al. and Thorsson et al. (**Table S11**) (46, 47). The results demonstrated that the activities of immune suppression and antigen processing were the highest in HAcluster_B, and the immune cytolytic activity of HAcluster_B was the lowest among the three groups, in agreement with previous analyses (**Figure 3F**).

### Construction of a Digital Model for Quantifying Histone Acetylation Patterns of Individual HCC Patients

**Figure 4:**
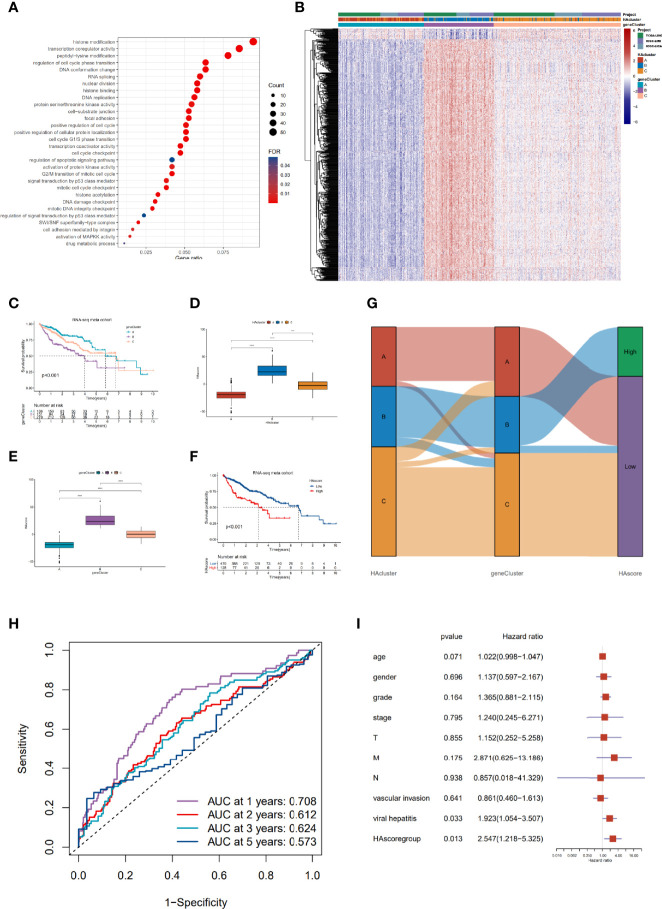
Construction of the characteristic signature of histone acetylation patterns and its prognostic significance. **(A)** GO enrichment analysis for histone acetylation pattern related genes with prognostic significance. The x-axis indicates the gene ratio within each GO term. **(B)** Unsupervised clustering of 591 histone-acetylation-related genes in RNA-seq meta cohort. The HAcluster, geneCluster, and cohorts were used as sample annotations. **(C)** The survival curves of different geneClusters in the RNA-seq meta cohorts (TCGA-LIHC and ICGC-LIRI) were estimated by the Kaplan–Meier plotter (p = 1.62e-05, Log-rank test). **(D)** Differences in the HAscores of the HAclusters in the RNA-seq meta cohorts. **(E)** Differences in the HAscores of the geneClusters in the RNA-seq meta cohorts. The statistical differences were tested by the Kruskal–Wallis test. (****P < 0.0001). **(F)** Survival analyses for low and high HAscore groups in the RNA-seq meta cohort (TCGA-LIHC and ICGC-LIRI) using Kaplan–Meier curves (*P* = 4.28e-07, Log-rank test). **(G)** Alluvial diagram demonstrating the changes in the HAcluster, geneCluster, and HAscore groups. **(H)** The predictive value of HAscore in patients from the TCGA-LIHC and ICGC-LIRI RNA-seq meta cohorts (AUC: 0.708, 0.612, 0.624 and 0.573 for 1, 2, 3, 5- year overall survival). **(I)** Multivariate Cox regression model analysis of the factors including HAscore, patient age, gender, TNM status, histology grade, vascular invasion, and viral hepatitis serologies in the TCGA-LIHC cohort.

To gain a comprehensive understanding of the differences in biological features among the three HAculsters, we identified 591 DEGs that were significantly associated with patient prognosis to characterize the HAcluster, based on three HAclusters previously analyzed in the RNA-seq meta cohort (**Figure S4A** and **Table S12**). The GO enrichment of these DEGs showed that their functions were mainly enriched in histone acetylation, cell cycle, RNA splicing, DNA replication, and cell adhesion (**Figure 4A**). We found that patients could be clustered into three phenotype-related subtypes based on these DEGs, named geneCluster_A, geneCluster_B, and geneCluster_C, (**Figure S4B**, **C**). Most DEGs were highly expressed in geneCluster_B, followed by geneCluster_C and geneCluster_A (**Figures 4B** and **S4D**). Most histone acetylation regulators were highly expressed in geneCluster_B (**Figure S4E**). The survival analyses showed that patient prognosis in geneCluster_B was the worst, as analyzed in the RNA-seq meta cohort and GEO meta cohort (**Figures 4C** and **S4F**). To depict and quantify the histone acetylation pattern of individual HCC patients using a convenient and precise method, we constructed a score model based on these phenotype-related DEGs. This model was termed the histone acetylation score (HAscore; see *Materials and Methods*). We found that the HAscore was positively correlated with the mRNA expression of histone acetylation regulators and phenotype-related DEGs. The HAscore in HAcluster_B and geneCluster_B was the highest. The HAscore was moderately high in HAcluster_C and geneCluster_C, and the lowest in HAcluster_A and geneCluster_A (**Figures 4D, E**). Next, we divided patients into high HAscore and low HAscore groups using the Survminer package and conducted an overlap analysis of these three different classifiers based on a histogram of frequency distribution (analyzed on samples in the RNA-seq meta cohort with prognostic information). The results showed that samples in the high HAscore group were all from geneCluster_B (172 out of 204: 84.3%), while 166 out of 191 (86.9%) samples in HAcluster_B composed the majority of geneCluster_B. In addition, most of the patients in geneCluster A and geneCluster_C belonged to HAcluster_A and HAcluster_C, respectively, and contributed to the main part of the low HAscore group (**Figure 4G**). The above results suggest that these three computational methods of classification have a high degree of coincidence.

Furthermore, we analyzed the prognostic prediction value of the HAscore in patients with HCC. The results demonstrate that the patients in the RNA-seq meta cohort and GEO meta cohort with low HAscores, had a prominent survival benefit (**Figures 4F** and **S4G**). Based on the RNA-seq meta cohort, the AUCs of the time-dependent ROC curves for the HAscore were 0.708, 0.612, 0.624 and 0.573 at 1-, 2-, 3- and 5- year overall survival, respectively (**Figure 4H**). Similar results were obtained from the GEO cohort (**Figure S4H**). Next, we performed multivariate Cox regression analysis using patient clinical characteristics including age, sex, histologic grade, TNM stage, vascular invasion, and viral infection. We found that the HAscore was a robust and independent prognostic biomarker for evaluating outcomes of patients in the TCGA-LIHC and GSE14520 cohorts (**Figure 4I**, HR = 2.547, 95% CI: 1.218-5.325, *P* = 0.013; **Figure S4I**, HR = 1.647, 95% CI: 1.058-2.563, *P* = 0.027). In addition, survival analyses based on the HAscore were also conducted for stomach adenocarcinoma, bladder urothelial carcinoma, skin cutaneous melanoma, and head and neck squamous cell carcinoma. The results show that the survival prognosis of patients with high HAscores was worse than those of patients with low HAscores (**Figure S4J**). These results indicate that the HAscore was closely related to prognosis and could be seen as a risk factor for HCC and several other cancers.

### Clinical Features, Transcriptional Molecular Characteristics, and TME-Infiltrating Cells Associated With the HAscore

**Figure 5:**
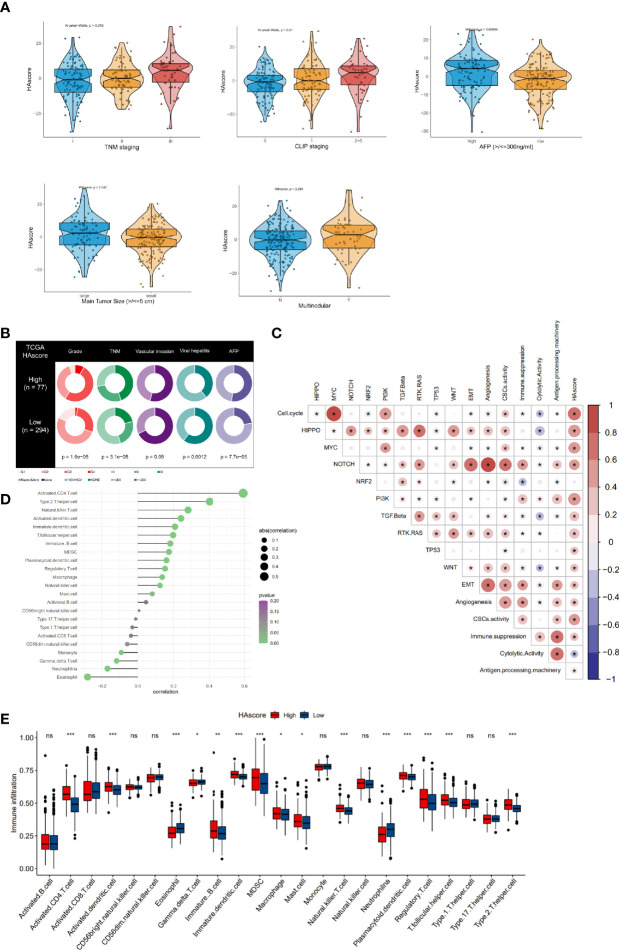
Clinical features, molecular characteristics, and TME infiltrating cells of the distinct HAscore groups. **(A)** Difference in HAscore among distinct clinical features related subgroups in the GSE14520 cohort. The Wilcoxon test was used to test the statistical differences among clinical features related subgroups. **(B)** Clinical features for the high and low HAscore groups in TCGA-LIHC cohort. Chi-squared test or Fisher test was used to test the statistical differences. **(C)** Correlations between the HAscore and the known gene signatures in RNA-seq meta cohort using Spearman analysis. Positive correlation is marked with red and negative correlation with blue. The asterisks represent the statistical *P* value (*P < 0.05). **(D)** Correlations between HAscore and TME infiltrating cell abundance in RNA-seq meta cohort using Spearman analysis. The circle size and x-coordinates describe the correlation coefficient. The color of the circle is scaled by *P* value. **(E)** Boxplot of each TME infiltrating cell abundance for high and low HAscore groups in the RNA-seq meta cohort. The statistical differences among the HAscore groups were tested by the Kruskal–Wallis test. (*P < 0.05; **P < 0.01; ***P < 0.001; ns, non-significant).

Our analyses have revealed survival prognostic differences between the high HAscore and low HAscore groups. Therefore, we determined to further explore the latent mechanism behind these results. We analyzed the relationship between the HAscore and the characteristics of the sample including clinical characteristics, transcriptional molecular background, and TME. The GSE14520 dataset and the TCGA-HCC cohort with adequate clinical information were used to analyze the correlation between HAscore and clinical characteristics. As shown in **Figures 5A** and **S5C**, the HAscore was higher in the groups with high AFP expression, vascular invasion, viral infection, multiple nodules, advanced histologic grade, TNM staging, and CLIP staging. In the TCGA-LIHC cohort, samples with high AFP expression, viral infection, vascular invasion, advanced histologic grade, and TNM staging were significantly higher in the high HAscore group (**Figure 5B** and **Figure S5D**). In the GES14520 dataset, samples with high AFP expression, advanced TNM staging, and CLIP staging were significantly higher in the high HAscore group (**Figure S5A, B**). Considering that the above-mentioned clinical characteristics were all risk factors for HCC prognosis (3, 61, 62), these results elucidate the fact that patients with a high HAscore had a worse survival prognosis.

Furthermore, the correlation between HAscore and tumor molecular background was analyzed. The results show that nearly all the cancer-related malignant pathways (such as cell cycle, HIPPO, MYC, PI3K, and MYC), excluding the NRF2 signaling pathway, were significantly positively correlated with the HAscore (**Figure 5C** and **Table S13**). The EMT score was also higher in the high HAscore group (**Figure S5E**), indicating that patients with high HAscores had higher activation of the malignant pathway, resulting in a worse prognosis. Next, correlation analysis involving HAscore, tumor-infiltrating immune cells, and immune function was performed (**Figure 5D**). The results demonstrate that the infiltration of pro-tumorigenesis cells, type 2 T-helper cells (*P* = 1.5e-13), and immunosuppressive cells, including MDSCs (*P* = 6.1e-05) and regulatory T cells (*P* = 0.00099), were significantly positively correlated with the HAscore. The immune cytotoxic cells-gamma delta T cells that were significantly negatively correlated with the HAscore (*P* = 0.02026). The HAscore was also significantly positively correlated with the activity of immune suppression (*P* = 4.536376e-12) and negatively correlated with immune cytolytic activity (*P* = 1.827941e-09) (**Figure 5C**). Additionally, in the high HAscore group the enrichment of the number of MDSC, regulatory T-helper cells, and type 2 T-helper cells was significantly higher, whereas that of the number of cytolytic gamma delta T cells was significantly lower (**Figure 5E**). The above results demonstrate that the HAscore was closely correlated with TME, and the high HAscore group was considered an immunosuppressive subtype.

### The Predictive Ability of the HAscore Model in the Sensitivity of Anti-Tumor Drugs

**Figure 6:**
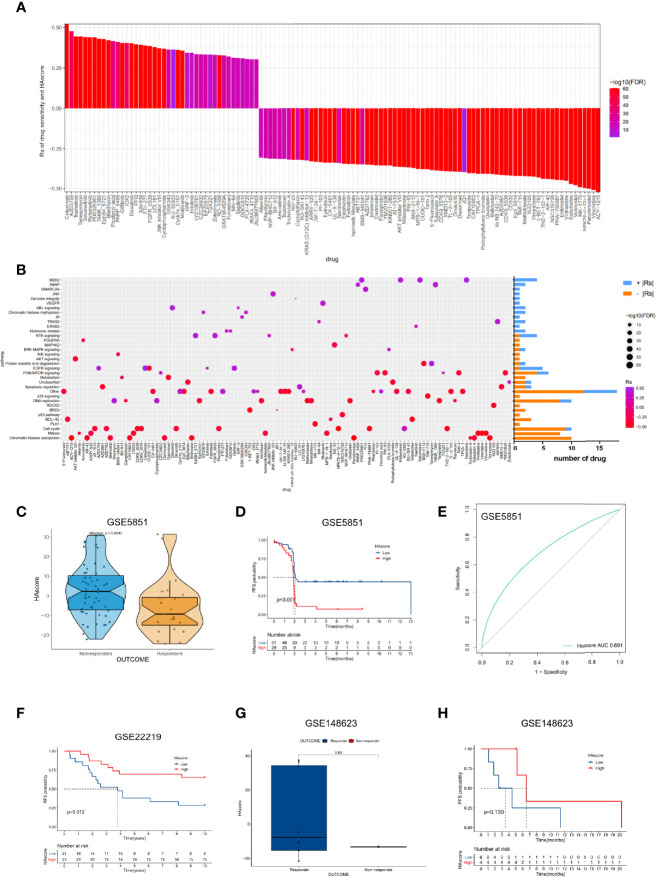
The relationship between HAscore and drug sensitivity. **(A)** The Spearman analysis was used to evaluate the correlation between HAscore and AUC of drug-sensitive curve. The brightness of column indicates the significance of the correlation. The height indicates the values of Rs. **(B)** Signaling pathways targeted by drugs that were closely correlated with HAscore. The horizontal axis shows the drug names, and the vertical axis shows the signaling pathway targeted by the drugs. The bar graph on the right displays the number of drugs in each signaling pathway. The significance of the correlation is shown by the size of the point. **(C, G)** The difference of HAscores between distinct clinical outcomes of related anti-tumor drugs, including cetuximab **(C)** and ricolinostat **(G)**. **(D, F, H)** Kaplan–Meier curves show the overall survival time in high HAscore or low HAscore group after the treatment of related anti-tumor drugs, including cetuximab **(D)**, a cyclophosphamide, methotrexate, and 5-fluorouracil regimen **(F)**, and ricolinostat **(H)**. **(E)** The predictive value of the HAscore to the sensitivity of cetuximab (AUC = 0.691).

Recently, numerous molecular-targeted agents have been developed for the treatment of certain cancers and have had good results. The above analyses reveal that histone acetylation modification is closely related to the functional pathways of cancer, such as cell cycle, DNA replication, the p53 pathway, and the PI3K/mTOR signaling pathway. Thus, the HAscore could have potential value in predicting the related drug response in patients. To test this hypothesis, we assessed the association between the HAscore and the response to drugs in cancer cell lines using the GDSC database. Using the Spearman correlation analysis, we identified 42 correlated pairs in which the AUC of the drug-sensitive curve was significantly positively correlated with HAscore (**Table S14**). These drugs included cetuximab, a monoclonal antibody that inhibits epidermal growth factor receptor (Rs = 0.522, *P* < 3.15E-61), the MEK inhibitor trametinib (Rs = 0.444, *P* < 3.15E-61), and the HSP90 inhibitor tanespimycin (Rs = 0.443, *P* < 3.15E-61). These results suggest that these drugs could be more sensitive in samples with low HAscores. In contrast, 74 correlated pairs were identified in which the AUC of the drug-sensitive curve was significantly negatively correlated with HAscore. These included the HDAC6 inhibitor ACY-1215 (Rs = -0.521, *P* < 3.15E-61), Wee1 inhibitor MK-1775 (Rs = -0.492, *P* < 3.15E-61), and Bcl-2 inhibitor sabutoclax (Rs = -0.472, *P* < 3.15E-61). These results suggest that these drugs could be more sensitive in samples with high HAscores (**Figure 6A**). Additionally, the signaling pathways of the genes targeted by these drugs were analyzed. Notably, the drugs that were sensitive in samples with high HAscores mostly targeted histone acetylation, mitosis, cell cycle, and DNA replication. This result is consistent with our previous analyses, which demonstrated that most histone modification regulators were highly active in the high HAscore group, along with cell cycle and DNA replication. In addition, we found that the drugs that were sensitive in samples with low HAscores mostly targeted the MEK2 and RTK signaling pathways (**Figure 6B**).

To examine whether the HAscore could predict the drug response in patients, we analyzed the relationship between drug response and HAscore based on several datasets that were treated with related anti-tumor agents. In the GSE5851 dataset, an analysis of cetuximab monotherapy in patients with advanced metastatic colorectal cancer reveals that the HAscore of responders was significantly lower than that of non-responders (**Figure 6C**), and the progression-free survival (PFS) of the low HAscore group was significantly longer than that of the high HAscore group (**Figure 6D**). The AUC of drug sensitivity-dependent ROC curves for the HAscore was 0.691 (**Figure 6E**). These results are consistent with our finding that the sensitivity of cetuximab was higher in the low HAscore group. Furthermore, in the GSE22219 dataset, an analysis of a cyclophosphamide, methotrexate, and 5-fluorouracil regimen in patients with breast cancer shows that the PFS of patients with high HAscores was significantly longer (**Figure 6F**), consistent with our previous analyses, which showed that methotrexate (Rs = -0.422, *P* < 3.15E-61) and 5-fluorouracil (Rs = -0.386, *P* < 3.15E-61) were more sensitive in high HAscore samples. The above results indicate that ACY-1215 (ricolinostat), an HDACi, was sensitive in the high HAscore sample. The analysis based on the GSE148623 dataset reveals higher HAscores in responders and longer PFS in high HAscore patients (**Figures 6G, H**); however, this was not statistically significant because of the small sample size (*N* = 10). Collectively, these analyses indicate that the HAscore has potential value in predicting drug response in patients.

### The HAscore Model Predicts Response to Immunotherapy With a PD-L1 or PD-1 Blocker

**Figure 7:**
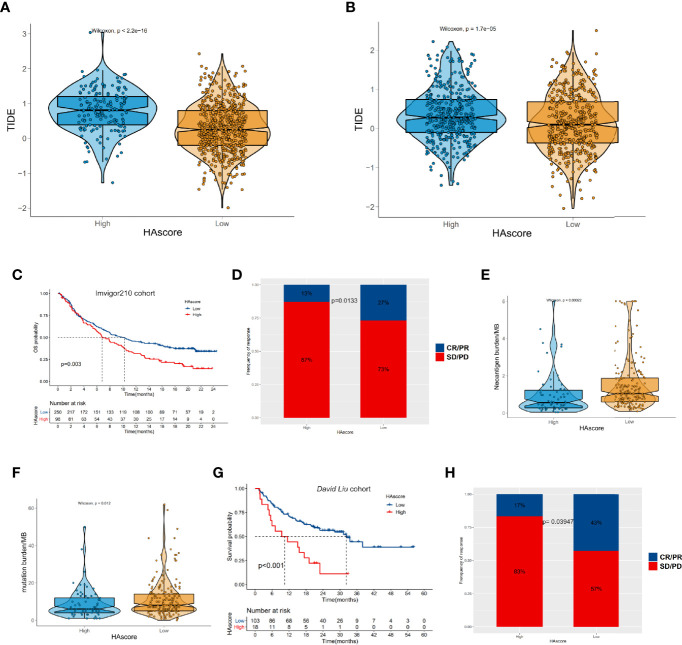
The relationship between HAscore and immunotherapy. **(A, B)** The TIDE scores of individual HCC samples in the high HAscore or the low HAscore groups. **(A)** shows the result from the RNA-seq meta cohort and **(B)** shows the result from the GEO meta cohort. **(C, G)** Kaplan–Meier curves show the overall survival time in the high HAscore or the low HAscore groups after the treatment of PD-L1 pathway blockgade immunotherapy **(C)** or PD-1 pathway blockade immunotherapy **(G)**. **(D, H)** The proportion of patients with different responses to PD-L1 blockage **(D)** or PD-1 blockage **(H)**. **(E, F)** the differences of neoantigen burden **(E)** or mutation burden **(F)** in the high HAscore or the low HAscore group.

The emergence of immunotherapies targeting the PD-L1 and PD-1 pathway blockade provides a positive outlook for patients with cancer. However, the benefits of ICI therapy are still limited because of innate or acquired immunotherapy resistance. Thus, many studies have aimed to identify predictors of ICI therapy for appropriate candidates, such as TIDE, which is widely used and strongly recommended to evaluate the immune response in cancer-related studies (63–68). Considering that the HAscore appears to be closely correlated with the TME, we examined the power of the HAscore to predict the response of patients to ICI therapy based on two immunotherapy cohorts. First, we analyzed the relationship between the HAscore and TIDE based on the TCGA-ICGC and GEO cohorts. The results show that the TIDE scores were significantly higher in the high HAscore group for both cohorts (*P <*2.2E-16; *P* = 1.7E-05; **Figures 7A, B**), and the HAscore was positively correlated with the TIDE score (Rs = 0.31; *P* < 2.2E-16; Rs = 0.15; *P* = 2.2E-05) (**Figures S6A, B**). In addition, the HAscore was significantly positively correlated with MDSC infiltration (Rs = 0.49; *P* = 1.37e-47; Rs = 0.67; *P* = 4.03e-109) and exclusion immune subtype (Rs = 0.46; *P* = 1.38e-42; Rs = 0.29; *P* = 1.05e-17) calculated by the TIDE method in TCGA-ICGC and GEO cohorts (**Figures S6C, D**). This result is consistent with our previous finding, which demonstrated that the high HAscore group was an immune suppressive subtype. Further, analysis in the anti-PD-L1 immunotherapy cohort (Imvigor210) shows that patients with a low HAscore had prolonged overall survival time (*P* = 0.003) (**Figure 7C**) and better therapeutic outcomes. The proportion of patients with complete response (CR) or partial response (PR) to the anti-PD-L1 blocker was 27% in the low HAscore group versus 13% in the high HAscore group (**Figure 7D**, chi-squared *P* = 0.0133). **Figures 7E** **, F** show that the neoantigen burden and mutation burden were high in the low HAscore group (*P* = 0.00022; *P* = 0.012), and the TIDE score was low in the low HAscore group. This is consistent with the finding that patients with low TIDE score seemed to gain more clinical benefit from IBI therapy (**Figure S6E**). **Figure S6F** shows that the AUC of the sensitivity-dependent ROC curve was 0.606 for the HAscore vs. 0.582 for TIDE score (*P* = 0.608). The study of the *David Liu* cohort that was treated with anti-PD-1 immunotherapy yielded similar results. **Figure 7G** shows that the OS of patients with low HAscores was significantly longer than that of patients with high HAscores (*P* < 0.001). Additionally, the proportion of patients with CR or PR to the anti-PD-1 blocker was 43% in the low HAscore group versus 17% in the high HAscore group (**Figure 7H**, Fisher; *P* = 0.03947). The above results indicate that patients with low HAscores could gain more survival advantage and greater benefit from ICI treatment. Further, the established modified histone acetylation score model could improve the selection of drugs for HCC and the prediction of response to anti-PD-L1 or anti-PD-1 immunotherapy.

## Discussion

Ample evidence exists showing that histone acetylation plays an essential role in cancer biological processes such as proliferation, apoptosis, differentiation, EMT, and drug sensitivity (69). Recently, researchers have found that histone acetylation also has an indispensable role in shaping the TME, which is an important factor in determining patient prognosis. However, most studies have focused on a single histone acetylation regulator. Relatively little is known about the relationship between the three types of histone acetylation regulators (“writer,” “eraser,” and “reader”) and their function in cancer. Considering that the histone acetylation regulators function as a tight network, it is necessary to analyze them as a whole in cancer research.

In this study, we analyzed the correlation among 36 histone acetylation regulators and found that the expression levels of nearly all of the regulators were positively correlated with each other; however, the functions of these regulators were different (even opposite). Based on unsupervised clustering of the 36 regulators, we divided the patients into three histone acetylation phenotypes (HAcluster_A, HAcluster_B, and HAcluster_C). Interestingly, their patterns were distinctly expressed in the 36 regulators. Nearly all the regulators had the highest expression in HAcluster_B, the regulators were moderately expressed in HAcluster_C, and the regulators had the lowest expression in HAcluster_A. This indicates that the activity and turnover of histone acetylation was intense in HAcluster_B. Our survival analysis reveals that the OS of patients in HAcluster_B was the worst of the three phenotypes. Furthermore, to better characterize the three histone acetylation phenotypes, we identified differentially expressed genes among them. Based on these genes, we constructed an HAscore model to digitally quantify the histone acetylation phenotype in individual patients. The results show that the HAscore was the highest in HAcluster_B, and the survival prognosis of the high HAscore group was the worst.

To explore the mechanism causing the prognostic difference among patients with different histone acetylation phenotypes, we first analyzed cancer biological features with the three histone acetylation patterns and two HAscore groups. We found that HAcluster_B was characterized by significant activation of the mTOR, ERBB, NOTH, WNT, TGF-β signaling pathways, cell cycle, and apoptosis. The HAscore was also significantly positively correlated with the activation of cell cycle, angiogenesis, EMT, cell stemness, and cancer-related malignant signaling pathways (HIPPO, MYC, NOTH, PI3K, TGF-β, RTK/RAS, TP53, and WNT). The above-mentioned biological functions and signaling pathways play an important role in promoting tumor development. For example, HIPPO (70), NOCTH (71), TGF-β (72) and WNT (73) are crucial signaling pathways that regulate various cancer-related processes, including cell proliferation, invasion, metastasis, and immunologic escape. The abnormal activation of these signaling pathways promotes cancer malignancy and leads to a poor prognosis (74–77).

Cancer stem cells are a subtype of cells that can self-renew by division and generate tumor progeny required for sneaking through and tumorigenesis (78, 79). In addition to their cancer-initiating ability, CSCs play a critical role in modulating other processes such as EMT (80), immunotherapy resistance (81) and drug resistance (82). These four signaling pathways also play key roles in supporting CSC activity (83). In HAcluster_B and the high HAscore group, where the malignant signaling pathways were active; the biomarkers for HCC stem cells were all highly expressed, indicating the high activity of CSCs in these two groups. These findings can partially explain why patients in HAcluster_B or those with high HAscores had the worse survival prognosis.

ICI therapy is a potentially good application in this setting because it mobilizes the autoimmune system to kill cancer cells. Mounting evidence has confirmed that diverse HDACi could alter the biological processes of immune cells and reshape the immune microenvironment, enhancing the tumor-killing effect of the immune system (84–86). In this study, we found that histone acetylation patterns were closely related to TMEs, and there were distinct differences in tumor-infiltrating immune cells among the three histone acetylation patterns. The activated dendritic cells, plasmacytoid dendritic cells, and antigen processing activity were significantly higher in HAcluster_B and the high HAscore groups. The biological processes of antigen processing and presentation play a critical role in improving the cancer-killing effect of immune cells (87). Previous studies have pointed out that HDACi, which improve the level of histone acetylation, could enhance antigen presentation by cancer cells (26, 85, 88). Interestingly, HAcluster_B and the high HAscore group had the highest expression of HATs, which improves histone acetylation levels, and this could be the reason for the high antigen processing and presentation observed in these two groups. Future research will have to confirm this hypothesis. Although antigen processing and presentation are active in HAcluster_B and the high HAscore groups, the immune-suppressive cells, MDSCs, and regulatory T cells were higher in both of them. This indicates that the HAcluster_B and the high HAscore groups were immune-suppressive subtypes, and the pro-immunity effect brought by activated antigen processing and presentation was offset by the immune-suppressive cells. Further functional enrichment analysis confirmed that HAcluster_B was highly enriched in immunosuppressive gene signatures and less enriched in immune cytolytic gene signatures. In addition, the HAscore was positively correlated with immune suppression and negatively correlated with cytolytic activity. These analyses indicate that the immune-suppressive subtype may be a reason for the poor prognosis of patients in the HAcluster_B group or with a high HAscore.

Finally, considering the strong relationship between histone acetylation patterns, cancer-related malignant signaling pathways, and TME, we examined the potential therapeutic effects of the HAscore. We found that it was positively correlated with the sensitivity of drugs targeting histone acetylation, cell cycle, mitosis, DNA replication, BRD3, and ROCK2. In contrast, we found that the HAscore was negatively correlated with the sensitivity of drugs targeting MEK2, PARP, VEGFR, ABL signaling, and histone methylation. These results imply that patients with higher HAscores could benefit more from the positively-correlated drugs while the negatively correlated drugs would be more suitable for patients with lower HAscores. In addition, we found that the HAscore could also predict the response of patients to anti-PD-L1 or anti-PD-1 immunotherapy. Compared to the patients with high HAscores, patients with lower HAscores were more sensitive to ICI immunotherapy. However, the benefits of ICI treatment are still limited due to the primary, adaptive, and/or acquired resistance to cancer immunotherapy (14). Fortunately, researchers have found that certain molecular-targeted anti-tumor agents can prevent cancer’s immunotherapy resistance and combining these anti-tumor agents with ICI immunotherapy could greatly improve patient prognosis rather than a single-drug regimen. For example, researchers have found that the combination of a selective HDAC3 inhibitor with anti-PD-L1 immunotherapy enhanced tumor regression in a syngenic murine lymphoma model (86). Additionally, a phase 2 clinical trial has shown that camrelizumab (a PD-1 monoclonal antibody) combined with apatinib (a VEGFR-2 tyrosine kinase inhibitor) shows promising efficacy and acceptable safety in patients with advanced HCC in both the first-line and second-line settings (89). This result is significantly better than ICI therapy using a single immune-checkpoint inhibitor (90, 91). Our findings provide evidence that the HAscore can be a predictor for the sensitivity of certain targeted drugs combined with ICI therapy. This indicates that there are potential new treatment options for choosing a suitable targeted agent to improve the outcome of immunotherapy in patients with HCC.

## Conclusion

In this study, we comprehensively evaluated the histone acetylation patterns of 1599 HCC cancer samples based on 36 histone acetylation regulators and identified three distinct histone acetylation patterns. The integrated analysis indicates that the differences in the activation of cancer-related malignant pathways and TME could be the main reason for the distinct prognostic outcomes of the three histone acetylation patterns. Based on the transcriptional differences among histone acetylation phenotypes, we constructed an HAscore model to digitally depict them, and identified the therapeutic utility of the HAscore in targeted therapy and immunotherapy. In summary, our study shows that evaluating the histone acetylation patterns of individual tumors will enhance our understanding of the characteristics of the TME and help develop personalized, combined, and immune-targeted therapeutic strategies for HCC patients. However, there are limitations in this study. The prognostic value of HAscore model on five-year OS of HCC patients is unsatisfactory. In future, more efforts should be paid to improve this model.

## Data Availability Statement

The datasets presented in this study can be found in online repositories. The names of the repository/repositories and accession number(s) can be found in the article/**Supplementary Material**.

## Ethics Statement

Written informed consent was obtained from the individual(s) for the publication of any potentially identifiable images or data included in this article.

## Author Contributions

Conception and design: MP and DY. Development of methodology: MP, DY, WL, and YX. Acquisition of data: WL and YX. Analysis and interpretation of data (e.g., statistical analysis, bioinformatic, computational analysis):YX and WL. Writing, review, and/or revision of the manuscript: WL, YX, QL, DY, and MP. Administrative, technical, or material support: MP and DY. Study supervision: YX, WL, MP, and DY. All authors contributed to the article and approved the submitted version.

## Funding

This research was supported by a grant from the National Natural Science Foundation of China (No.82072627, No.81872385) and Natural Science Foundation of Guangdong Province of China (No.2016A030313626).

## Conflict of Interest

The authors declare that the research was conducted in the absence of any commercial or financial relationships that could be construed as a potential conflict of interest.

## Publisher’s Note

All claims expressed in this article are solely those of the authors and do not necessarily represent those of their affiliated organizations, or those of the publisher, the editors and the reviewers. Any product that may be evaluated in this article, or claim that may be made by its manufacturer, is not guaranteed or endorsed by the publisher.

## Abbreviations

HCC, hepatocellular carcinoma; MDSC, myeloid-derived suppressor cells; TME, myeloid-derived suppressor cells; HBV, hepatitis B virus; HCV, hepatitis C virus; TACE, transarterial chemoembolization; ICI, immune-checkpoint inhibition; PTM, posttranslational modification; HATs, histone acetyltransferases; HDACs, histone acetyltransferases; HDACi, histone deacetylases inhibitors; TCGA, the cancer genome atlas; ICGC, international cancer genome consortium; GEO, gene expression omnibus; AFP, alpha-fetoprotein; OS, overall survival; DEGs, differentially expressed genes; PFS, progression-free survival; CR, complete response; PR, partial response; SD, stable disease; PD, progressive disease; CMF, 5-fluorouracil; EMT, epithelial-to-mesenchymal transition; CSCs, cancer stem cells; GSVA, gene set variation analysis; ssGSEA, single-sample gene-set enrichment analysis; GDSC, genomics of drug sensitivity in cancer; TIDE, the tumor immune dysfunction and exclusion; ROC, receiver operating characteristic; PCA, principal component analysis; HR, hazard ratio; CNV, copy number variation.
