# Supplementary material for: Histone Acetylation Regulator-Mediated Acetylation Patterns Define Tumor Malignant Pathways and Tumor Microenvironment in Hepatocellular Carcinoma
Source: Front Immunol. 2022 Jan 25;13:761046. doi: 10.3389/fimmu.2022.761046 (PMC8821108; doi:10.3389/fimmu.2022.761046)
Supplement: Supplementary file 1 [file DataSheet_1.pdf]

Supplementary Material

1 Supplementary Figures and Tables

1.1 Supplementary figures

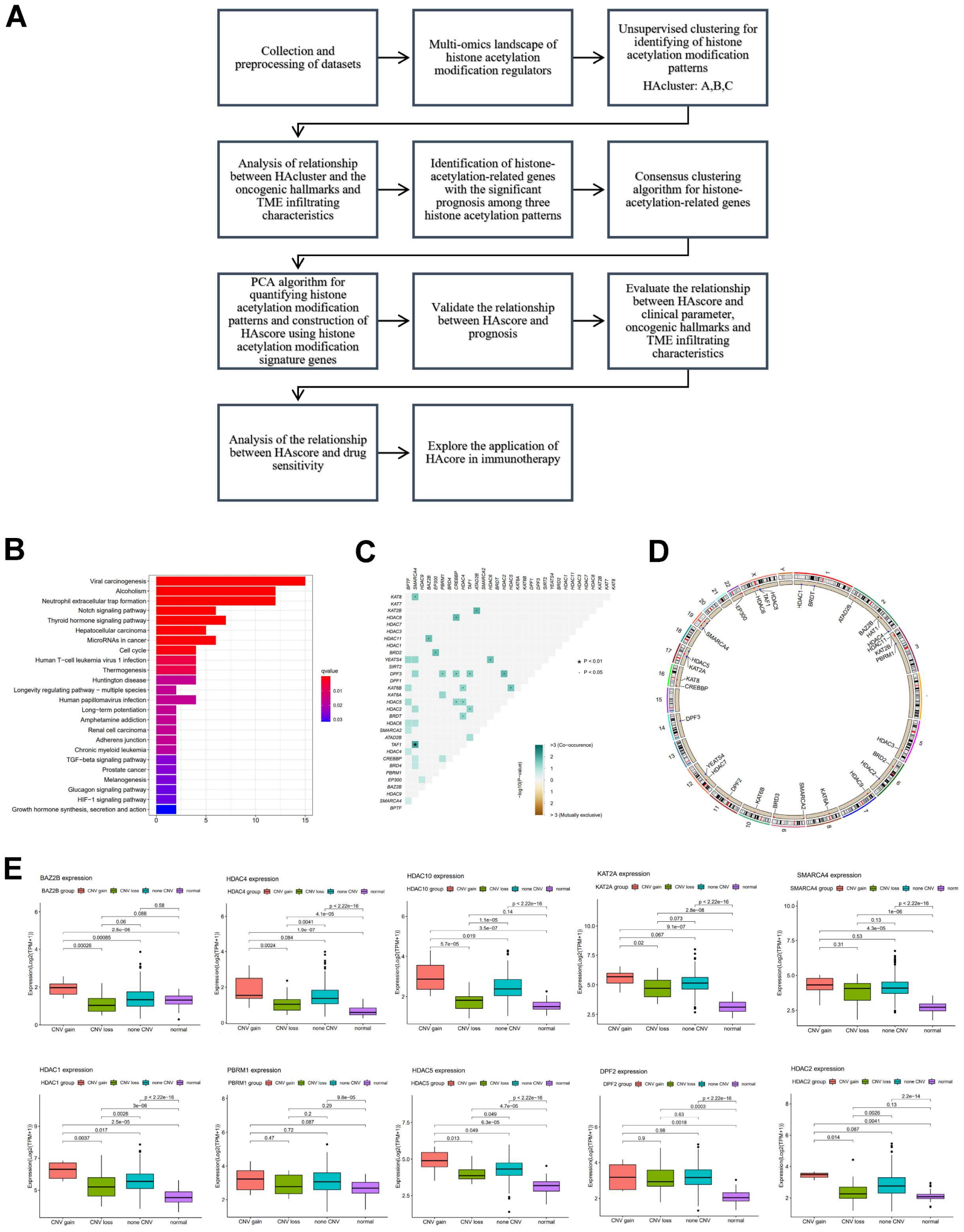

Supplementary Figure 1. Overview of study design and analysis of mutation frequency and copy number variations (CNVs) in TCGA-LIHC. **(A)** Study design and flowchart of the steps in the performed analyses. **(B)** KEGG enrichment analysis of 36

histone acetylation modification regulators. The x-axis indicates gene numbers enriched in each GO term. **(C)** The mutation co-occurrence and exclusion analysis for the 36 histone acetylation modification regulators in the TCGA-LIHC cohort. Co-occurrence: aquamarine; exclusion: claybank. **(D)** The location of CNV alteration of histone acetylation regulators on 23 chromosomes in the TCGA-LIHC cohort. **(E)** The expression of histone acetylation modification regulators among CNV alteration groups in the TCGA-LIHC cohort. The Wilcoxon test was used to estimate the statistical difference.

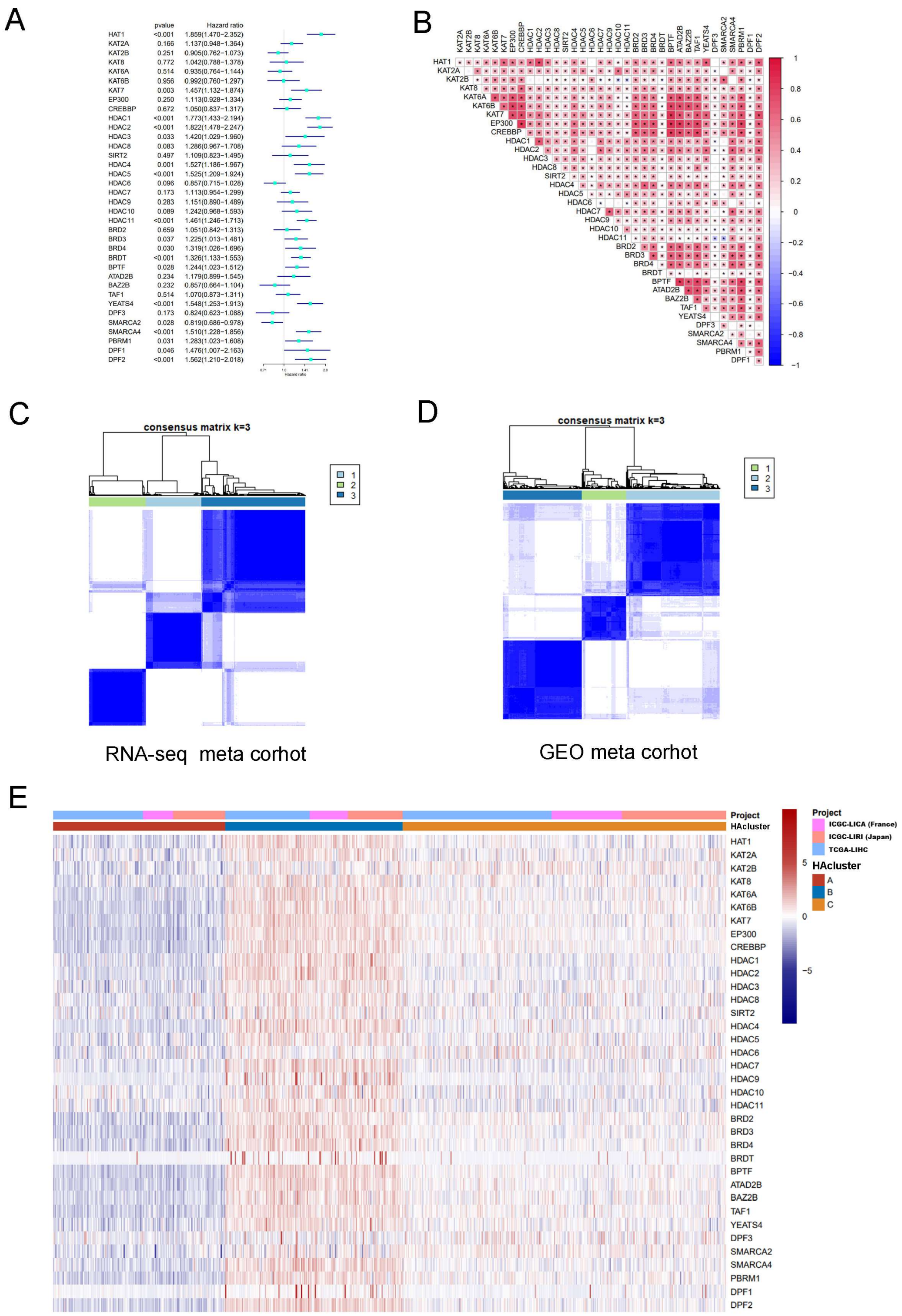

Supplementary Figure 2. Correlation and prognostic analysis of the 36 histone acetylation regulators and unsupervised clustering on HCC cohort. **(A)** Subgroup analysis estimating the prognostic significance of regulators by univariate Cox regression. **(B)** The correlations analysis among the 36 histone acetylation regulators in the RNA-seq meta cohort. Negative correlation: blue; positive correlation: red (**P* < 0.05). **(C, D)** Consensus clustering of the 36 histone acetylation regulators matrix

for k = 3 of 774 patients in the TCGA-seq meta cohort (TCGA-LIHC, ICGC-LIRI, ICGC-LICA) **(C)** and 825 patients in the GEO meta cohort (GSE14520, GSE76427, GSE116174, GSE104580, GSE112790, GSE121248) **(D)**. **(E)** Unsupervised clustering of the 36 histone acetylation regulators in the RNA-seq cohort [TCGA-LIHC, ICGC-LIRI (Japan), ICGC-LICA]. The HAclusters and project of database were used as sample annotations.

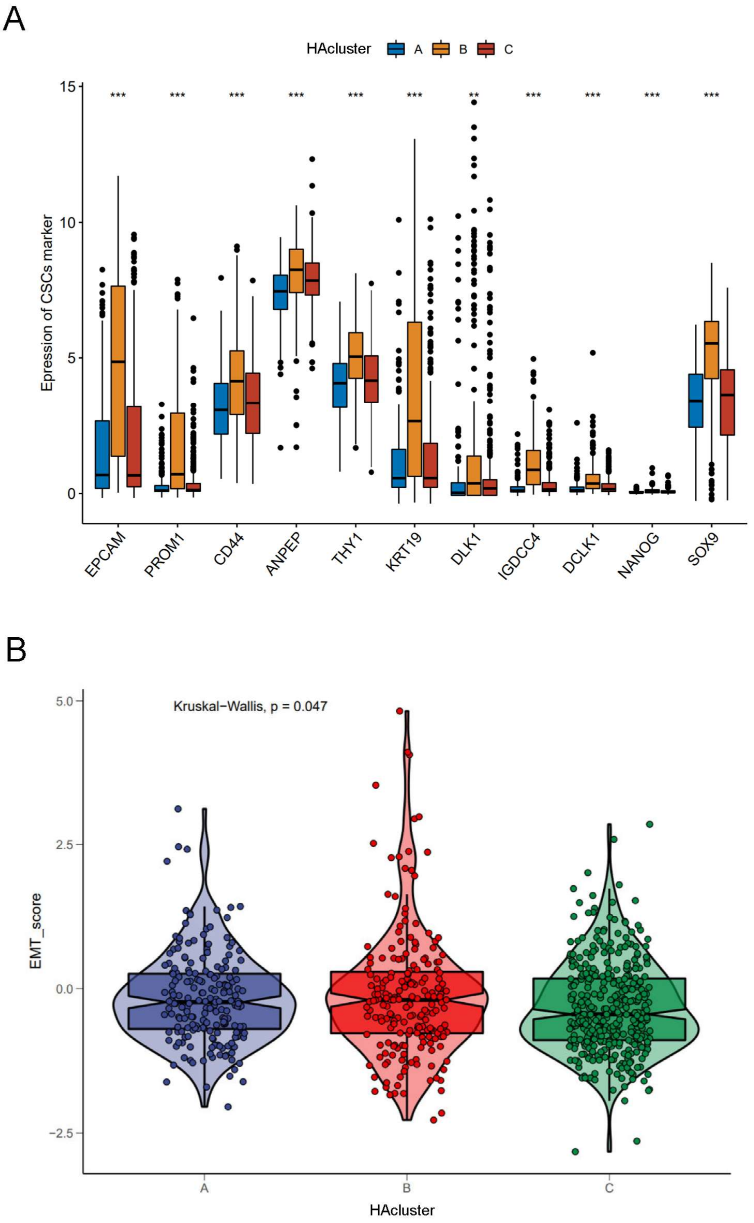

Supplementary Figure 3. Relationship between the HAclusters, EMT scores, and CSC markers in the RNA-seq meta cohort. **(A)** Differences in the expression of CSC markers among the three distinct HAclusters. **(B)** The difference in EMT scores for the three distinct HAclusters. The statistical differences among the three HAclusters were tested by the Kruskal–Wallis test. (**P* < 0.05; ***P* < 0.01; ****P* < 0.001).

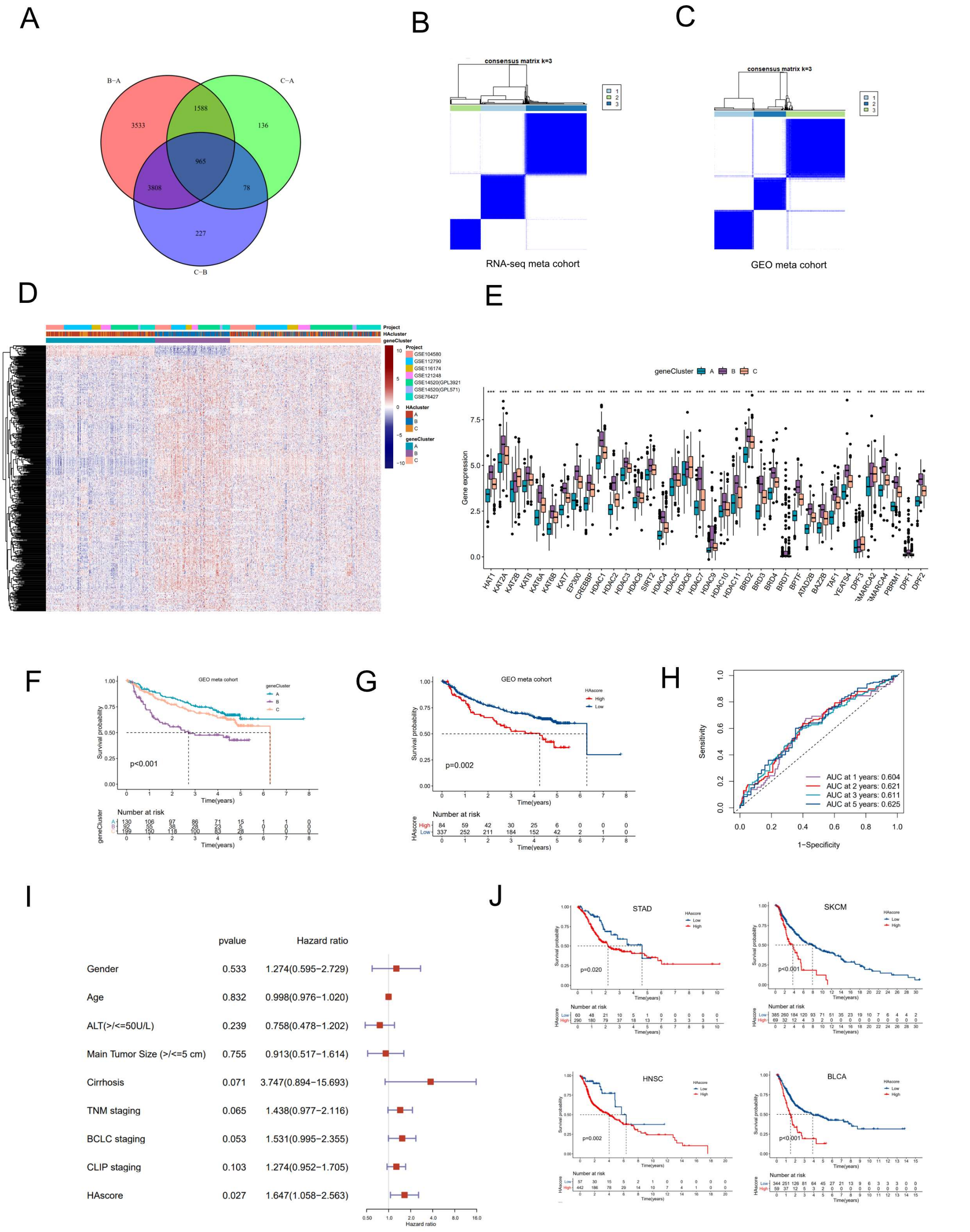

Supplementary Figure 4. Construction of the characteristic signature of histone acetylation patterns and prognostic significance. **(A)** Histone acetylation patterns (965) and related DEGs between the three HAclusters are shown in the Venn diagram. **(B, C)** Consensus clustering of 591 histone acetylation-related DEGs (associated with prognosis) matrix for k = 3 of 774 patients in the TCGA-seq meta cohort (TCGA-LIHC, ICGC-LIRI, ICGC-LICA) **(B)** and 825 patients in the GEO meta cohort (GSE14520, GSE76427, GSE116174, GSE104580, GSE112790, GSE121248) **(C)**. **(D)** Hot plot of 591 histone acetylation-related genes in the GEO meta cohort. The HAclusters, geneClusters, and cohorts were used as sample annotations. **(E)** The

expression of the 36 histone acetylation regulators in the three geneClusters. The asterisks represent the statistical *P* value (**P* < 0.05; ***P* < 0.01; ****P* < 0.001). The Kruskal–Wallis test was used to test the statistical differences among three geneClusters. **(F)** The survival curves of different gene clusters in the GEO meta cohort (GSE14520, GSE76427, GSE116174) were estimated by the Kaplan–Meier plotter (*P* = 0.0002, Log-rank test). **(G)** Survival analyses for low and high HAscore groups in the GEO meta cohort (GSE14520, GSE76427, GSE116174) using Kaplan–Meier curves (*P* = 3.43e-05, Log-rank test). **(H)** The predictive value of the HAscore in patients among the GEO meta cohort (GSE14520, GSE76427, GSE116174; AUC: 0.604, 0.612, 0.611 and 0.625; 1-, 2-, 3- and 5- year overall survival). **(I)** Multivariate Cox regression model analysis of the factors including HAscore, patient age, gender, TNM status, BCLC status, CLIP status, main tumor size, cirrhosis, and ALT in the GSE14520 cohort. **(J)** Survival analyses for low and high HAscore groups in the the Cancer Genome Atlas (TCGA)-stomach adenocarcinoma (STAD), TCGA-skin cutaneous melanoma (SKCM), TCGA-head and neck squamous cell carcinoma (HNSC), and TCGA-bladder urothelial carcinoma (BLCA) cohorts.

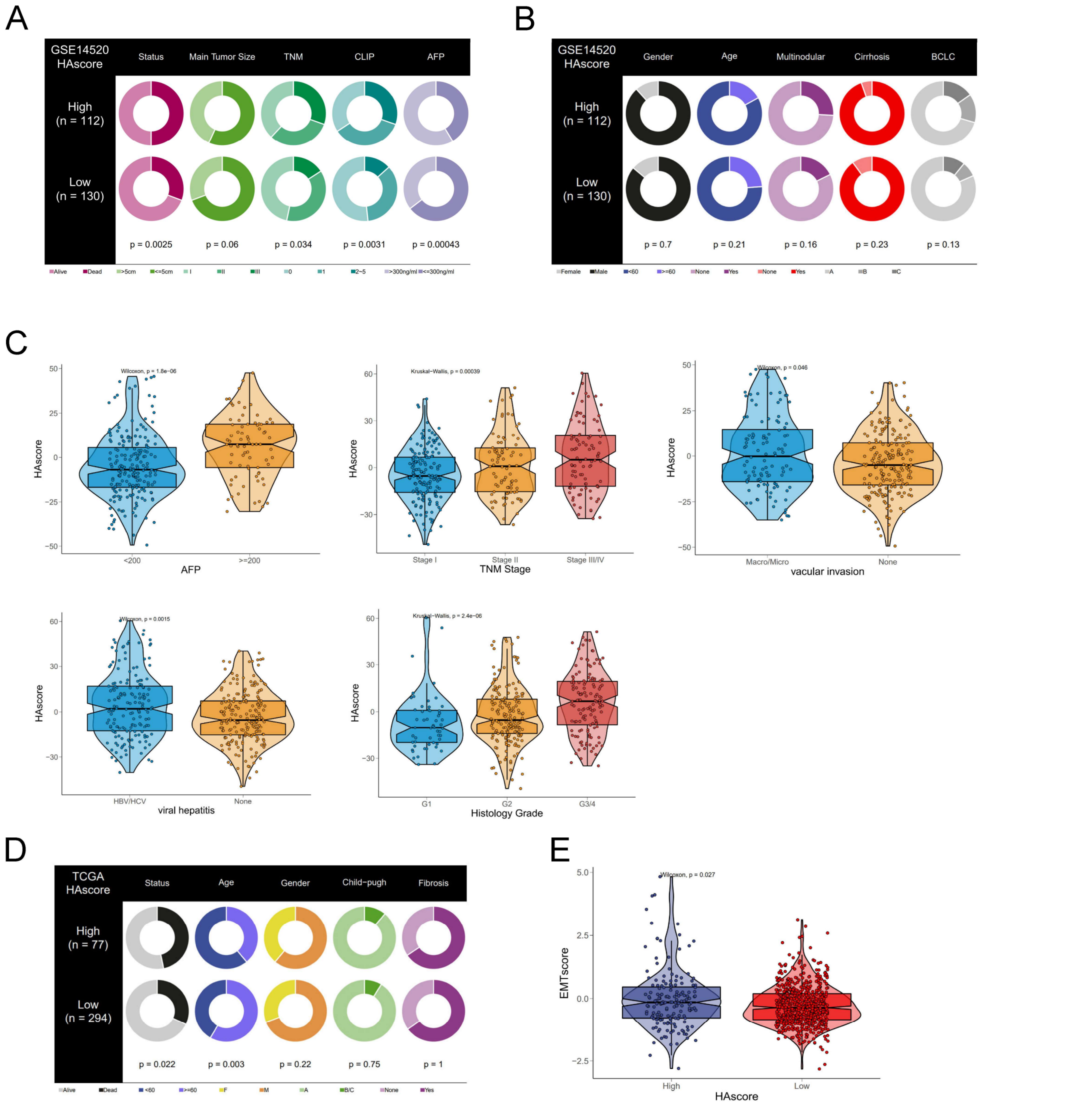

Supplementary Figure 5. Clinical features, molecular characteristics, and TME infiltrating cells of the distinct HAscore groups. **(A, B)** Clinical features for the high and low HAscore groups in the GSE14520 cohort. Chi-squared or Fisher was used to test the statistical differences. **(C)** Difference in HAscore among distinct clinical features subgroups in the TCGA-LIHC cohort. The Wilcoxon test was used to test the statistical differences among clinical features subgroups. **(D)** Clinical features for the high and low HAscore groups in TCGA-LIHC cohort. Chi-squared or Fisher was used to test the statistical differences. **(E)** The difference in EMT scores in high HAScore (purple) and low HAscore (red) groups in the RNA-seq meta cohort.

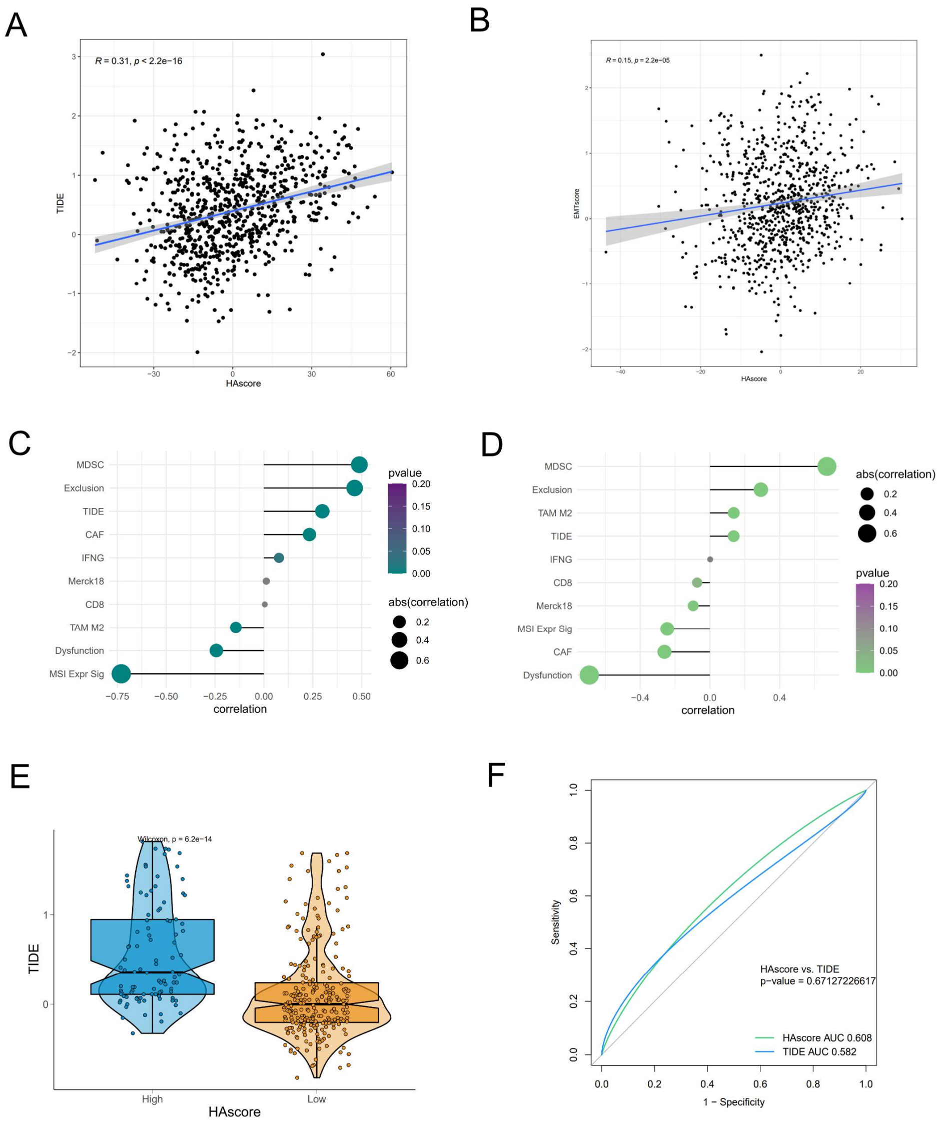

Supplementary Figure 6. The relationship between HAscore and TIDE immune evaluation. **(A, B)** The correlation analyses between TIDE score and HAscore for the RNA-seq meta cohort (Rs = 0.31, *P* < 2.2E-16) **(A)** and the GEO meta cohort (Rs = 0.15, *P* = 2.2E-05) **(B)**. **(C, D)** The correlation analyses between HAscore and immune features calculated by the TIDE method, based on the RNA-seq meta cohort **(C)** and the GEO meta cohort **(D)**. The brightness of the points indicates the significance of the correlation and the size of the points indicate the values of correlation coefficient. **(E)** The different TIDE scores in the high HAscore and the low HAscore groups: results from Imvigor210 cohort. **(F)** The comparison of the predictive value of the HAscore (AUC = 0.608) or the TIDE score (AUC=0.582) to the sensitivity of PD-L1 pathway blockade in patients from the Imvigor210 cohort.
